# A bibliometric evaluation and visualization of global solar power generation research: productivity, contributors and hot topics

**DOI:** 10.1007/s11356-023-31715-x

**Published:** 2024-01-04

**Authors:** Xiaozan Lyu, Tianqi Ruan, Wujun Wang, Xiaojing Cai

**Affiliations:** 1 Department of Administrative Management, School of Law, Hangzhou City University, Hangzhou, 310015 China; 2 Department of Energy Technology, KTH Royal Institute of Technology, 100 44, Stockholm, Sweden; 3 Research Center for Government Governance and Public Policy, School of Business, Yangzhou University, Yangzhou, 225127 China

**Keywords:** Solar power generation, Bibliometric analysis, Science mapping, Scientific production, Hot topics

## Abstract

The demand for sustainable energy is increasingly urgent to mitigate global warming which has been exacerbated by the extensive use of fossil fuels. Solar energy has attracted global attention as a crucial renewable resource. This study conducted a bibliometric analysis based on publication metrics from the Web of Science database to gain insights into global solar power research. The results indicate a stable global increase in publications on solar power generation and a rise in citations, reflecting growing academic interest. Leading contributors include China, the USA, South Korea, Japan, and India, with the Chinese Academy of Sciences emerging as the most prolific institution. Multidisciplinary Materials Science, Applied Physics, Energy and Fuels, Physical Chemistry, and Nanoscience and Nanotechnology were the most used and promising subject categories. Current hot topics include the systematic analysis of photovoltaic systems, perovskite as a solar cell material, and focusing on stability and flexibility issues arising during photovoltaic-grid integration. This study facilitates a comprehensive understanding of the status and trends in solar power research for researchers, stakeholders, and policy-makers.

## Introduction

Conventional power generation technologies rely on fossil fuels, exert pressure on the environment and ecosystems, and may become untenable in the future due to the scarcity of resources (Zhang et al. 2022). With the growing awareness of sustainable development, most countries have implemented policies and targets concerning renewable energy, and 57 have set goals to achieve 100% renewable energy utilization (REN21 2018). Consequently, renewable energy options like solar power, characterized by abundant resources and fewer negative environmental impacts, have become an important discussion topic for governments and energy stakeholders worldwide (Chen et al. 2020).

Solar energy has emerged as a promising alternative to traditional sources of electricity generation. This is accomplished through harnessing solar irradiance with two fundamental technologies: photovoltaics (PV) and concentrating solar power (CSP) (Pazheri et al. 2014; Azad and Parvin 2022). PV technology utilizes the photoelectric effect of specific chemicals, particularly silicon, to convert sunlight directly into electricity. Conversely, CSP is a technique that concentrates and directs sunlight onto high-temperature receivers, subsequently converting solar energy into thermal energy (Achkari and El Fadar 2020; Chen et al. 2020). To encourage the development of solar energy–related industries, most countries have enacted a suite of policies and regulations promoting renewable resources and fostering stable and sustainable growth for the PV sector. These measures include feed-in-tariffs, portfolio standards, tax credits, pricing laws, production incentives, quota requirements, and trading systems (Solangi et al. 2011; Chen et al. 2020). For example, the USA has implemented various policies to promote renewable energy usage, including renewable portfolio standards and federal investment tax credits. Additionally, federal loan guarantees have been provided to support the deployment of various types of renewable energy projects in the USA (Mehos et al. 2016). China’s solar power research programs, investment subsidies, and operational subsidies are geared toward achieving a 27.5% share of renewable energy by 2050, supported through various programs, subsidies, and policies (Mir-Artigues et al. 2019). Implementing supportive policies and regulations advances the transition toward a sustainable, low-emission future centered on alternative energy sources.

Since the 1970s, academic research on solar energy technologies has gained significant prominence due to its potential as a sustainable energy source. Over the past two decades, this topic has experienced substantial growth and advancements (Dong et al. 2012). Most research has focused on technological innovations and applications, including efforts to enhance production plans to improve efficiency (Cohen et al. 1999; Bacher et al. 2009), the application of novel technologies aimed at maximizing economic benefits (Cai et al. 2010; Verma et al. 2012), and forecasting improvements to mitigate uncertainties (Kayal and Chanda 2013; Brancucci Martinez-Anido et al. 2016; Kawamoto and Guo 2018). These investigations form the scientific framework to advance solar energy research. Furthermore, they provide essential data to assist researchers in exploring the solar energy developmental status on a global and macroscopic scale.

As a well-established quantitative research method, bibliometric analysis plays a crucial role in studying the current state of scientific research and forecasting future trends (Hicks and Melkers 2013) and has been widely adopted in various domains (Hood and Wilson 2001). Bibliometrics utilizes publication counts, patents, or citations to assess and interpret scientific and technological advancements. It summarizes scientific activities, explores fundamental scientific endeavors, gauges technical capabilities, and assesses research performance over time (Van Raan 1997; Hicks and Melkers 2013; Zhang et al. 2013, 2014). Through bibliometric analysis, researchers and policymakers can obtain various internal and external characteristics of publications, identify research hotspots and trends, and provide scientific and rational guidance for research planning (Mao et al. 2015b).

To the best of the authors’ knowledge, bibliometric analysis has been infrequently employed in solar power research at this time. While publications exist, several have a limited scope and fail to provide a comprehensive overview of a specific functional aspect (Moro et al. 2018). Alternatively, they might be generalized, encompassing several renewable technologies (Chen et al. 2016; Yu et al. 2016; Wang et al. 2017a, b). Others are focused solely on research in specific regions or areas (Chen et al. 2016; Liang and Liu 2018). Some literature is outdated or covers a narrow time period, requiring further updates and supplementary work (Dong et al. 2012; Mao et al. 2015a; De Paulo and Porto 2017; Wang et al. 2017a). Consequently, there is a research gap regarding a comprehensive bibliometric analysis of solar power research.

This study evaluates solar power generation research over the past two decades comprehensively using bibliometric analysis and tools. The investigation encompassed multiple research perspectives, including scientific output, prolific countries and institutions, main subject categories and journals, and hot topics. Additionally, visualization tools reveal developmental trends in solar power research, providing guidance to researchers about related technical layouts and hotspots. The central research questions include identifying the publication trends, the countries, institutions, subject categories, and journals that contribute the most to solar power research, the directions and temporal evolution of solar power research, and which topics have gained attention.

This study facilitates a critical evaluation of solar power research and offers insights into academic publishing activities. Specifically, this research enhances the understanding of research trends in solar energy generation using bibliometric analysis, illuminating development patterns and research gaps. Additionally, visualization tools demonstrate current trends and how solar power research has evolved. This study contributes a deeper understanding of the academic landscape related to solar power generation, enabling researchers to identify limitations and opportunities for future research. This research is of interest to researchers, policymakers, and practitioners focusing on renewable energy research and development.

This study is structured as follows. In the “Methodology” section, the methodology, data sets, and indicators are defined. The “Results” section presents the main findings of the reviews and the results of additional analyses. Finally, the “Conclusion” section concludes the study and discusses the implications of the empirical results.

## Methodology

### Data and indicators

The present study utilized a bibliometric dataset extracted from the Science Citation Index Expanded (SCI-E) within the Web of Science (WoS) Core Collection database, made available by Clarivate Analytics. The SCI-E database, including over 8500 distinct academic journals across diverse disciplines, spanning from the natural sciences and engineering to technology and clinical medicine, has an extensive historical reach, with its earliest publications dating back to the year 1900.^1^

**Fig. 1:**
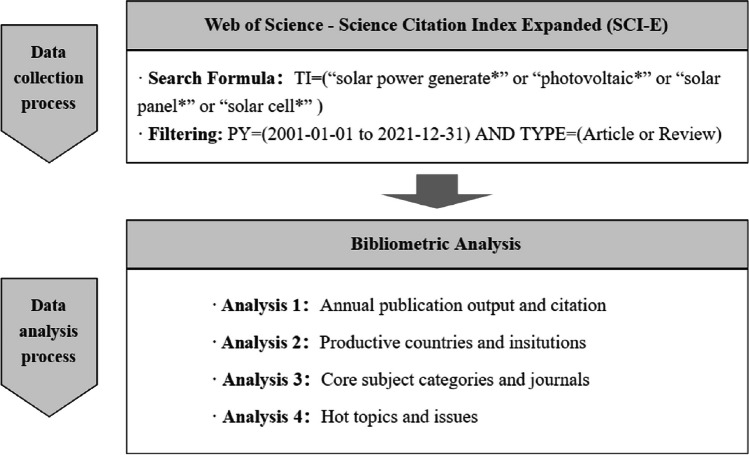
Data selection process and review methodology

The current study aimed to identify and analyze publications related to solar power generation from 2001 to 2021. Publication types, such as “Article” or “Review,” with terms, such as “solar power generate*,” “photovoltaic*,” “solar panel*,” and “solar cell*,” in the title field were retrieved using a bibliometric approach (Fig. 1). A total of 95,758 publications were obtained. Various bibliometric items, including publication year, journal, authors, addresses, author keywords, subject categories, and citation counts, were extracted and processed using specific algorithms. The data was analyzed and visualized using software, such as Microsoft Excel, Python, and VOSviewer (Van Eck and Waltman 2010). Not all publications related to the research topic of solar power generation were covered with our search strategy. However, a focused and precise approach is the most efficient for identifying publications with the most direct alignment with the core concept of “solar power generation.”

The data provided an overview of current research on solar power generation globally. This included the annual scientific output, academic performance of prolific countries and institutions, distribution of publications in subject categories and journals, and clusters of high-frequency title terms. To ensure data consistency and reliability, publications from England, Scotland, Ireland, and Wales were manually grouped under the UK heading. In addition, publications resulting from international collaborations, i.e., involving more than two authors from at least two countries, were extracted as a separate group to analyze the degree of internationalization of countries and institutions. To facilitate a rigorous and comprehensive analysis, a series of indicators were used as the main proxies for measuring the research productivity and impact. These indicators are listed in detail in Table 1, along with their corresponding definitions and calculations. By utilizing a diverse set of indicators, this study provides a comprehensive understanding of the research landscape in solar power generation.

**Table 1:** Indicators used in this study

Indicator	Definition
*TP*_*i*_	Number of publications in year *i*
*TP*	Total number of publications of a certain country or institution^a^
*TP%*	Percentage of publications of a certain country or institution in the global total
*ICP*	Number of international collaboration publications of a certain country or institution
*ICP%*	Percentage of internationally collaborated publications in the total publications of a certain country or institution (i.e., ICP/TP)
*CP*	Number of publications in a country or institution that received at least one citation
*CP%*	Percentage of publications that received at least one citation in the total publications of a certain country or institution (i.e., CP/TP)
*TC*	Total citations of publications of a certain country or institution
*Top-10%*	Percentage of top 10 highly cited publications in WoS of a certain country or institution, which is the *top 10%* most cited documents in a given subject category, year, and publication type divided by the total number of documents, displayed as a percentage^b^
*AC*	Average citations of publications of a certain country or institution (i.e., TC/TP)
*AAC*_*i*_	Annual average citation of each publication published in year *i*, the calculation is as follows: $AA{C}_{i}=\frac{TC}{TP\times (2022-i+1)}$

^a^Due to international co-authorship, the authors of a publication may come from more than one country or region, and when calculating the total number of publications (*TP*) for a country or region, the total number is added by one if the authors of the publication are affiliated with that country. Therefore, the same publication was counted in the total number of publications for different countries

^b^https://incites.help.clarivate.com/Content/Indicators-Handbook/ih-impact-indicators.htm

At the global level, the indicator of *TP*_*i*_ is the annual output of publications related to solar power generation, and *AAC*_*i*_ is the degree of attention obtained from the academic community. At the country and institution levels, multiple indicators were applied to evaluate and compare the academic performance of certain countries and institutions engaged in relevant research from three perspectives as follows:

- First, the *productivity*, including the number and percentage of publications (*TP* and *TP%*).
- Second, the *degree of internationalization* in research, reflected by the share of internationally co-authored publications (*ICP%*).
- Third, the *academic impact*, based on the proportion of cited publications (*CP%*) and top 10 percent highly cited publications (*Top-10%*), as well as the average citations (*AC*).

### Overlay maps and VOSviewer

This study introduced overlay maps in the scientific mapping field, building on previous work done by Boyack (2009) and further developing these ideas into interactive overlays (Bensman and Leydesdorff 2009; Leydesdorff and Rafols 2009; Rafols et al. 2009). An overlay map was constructed on a base map with the location (or clusters) of units under study to visualize the properties or features of such units (Rafols et al. 2009). This study generated a network of high-frequency title terms based on their co-occurrence in publications, creating the base map of hot topics using VOSviewer. The text mining functionality of VOSviewer supported the creation of term maps based on a corpus of documents, following these steps (Van Eck and Waltman 2011):

i. Identifying noun phrases using an approach developed by Van Eck et al. (2010a, b). The linguistic filter selected all word sequences that consisted exclusively of nouns and adjectives and ended with a noun, identifying noun phrases.
ii. Selecting the most relevant noun phrases, or terms. For each noun phrase, we determined the distribution of (second-order) co-occurrences over all noun phrases. A higher difference between the two distributions indicated greater relevance of a noun phrase. Subsequently, noun phrases with high relevance were grouped into clusters.
iii. Mapping and clustering of the terms. The unified framework for mapping and clustering (Van Eck et al. 2010a, b; Waltman et al. 2010) was used in this step.
iv. Visualization of the results obtained from mapping and clustering.

Visualizing research trends and characteristics is fundamental to scientific inquiry. VOSviewer, a software tool enabling the analysis and visualization of bibliometric data, is a valuable resource for researchers (Wong et al. 2021). VOSviewer has an overlay option using a network visualization that assigned colors to items based on their overlay scores. This allows the visualization of average publication year and citation counts of terms across the base map, providing an intuitive indication of the characteristics of different research topics. The overlay score in this context was defined as the average publication year and citation count of the high-frequency title terms. In the current study, the overlay employed a color range from white (low score) to red (high score).

## Results

Several analyses examined current research on global solar power generation. This section presents the results, including global scientific output, the most prolific countries and institutions, the main subject categories, journals, and hot topics that emerged from clusters of frequently used title terms. These analyses provide a comprehensive view of the existing knowledge landscape, enabling the identification of key focus areas and concerns for improved solar power generation.

### Global scientific output

**Fig. 2:**
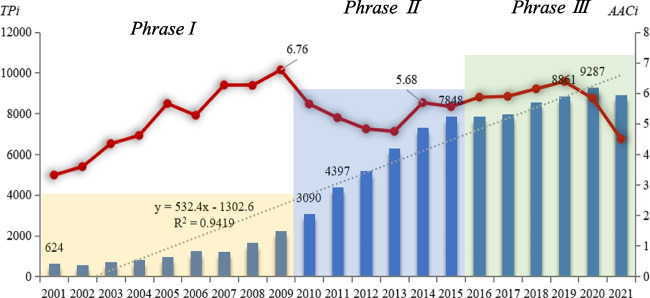
Annual output (*TP*_*i*_) and average citations *(AAC*_*i*_) of publications. The bars represent the number of total publications each year, dots represent the average citations of all publications in the corresponding year, and color blocks represent phases based on the annual number of publications and the growth rate

The present study examined the annual number of publications (*TP*_*i*_) and average citations (*AAC*_*i*_) related to solar power generation over twenty years (2001–2021), represented in Fig. 2 as bars and strip scatter, respectively. The annual output of global publications on solar power generation exhibited a linear trend (*R*^2^ > 0.94), indicating stable growth in academic interest. Approximately 2–5% of the annual publications from 2001 to 2020 have not been cited thus far. The proportion of publications not cited in 2021 is higher (approximately 10%) due to the more recent publication date and shorter citation window.

Depending on the annual number of publications and the growth rate, three distinct phases were identified and marked with different colors (yellow, blue, and green). The initial phase from 2001 to 2009 revealed a modest output of academic research in solar power generation, with approximately 1000 publications and a low growth rate around 15%. During the second phase, 2010–2015, the number of publications increased rapidly, with an annual growth rate of approximately 30%. In 2010, the number of publications exceeded 3000, and by 2015 exceeded 7000, representing a substantial increase. However, in the third phase since 2016, the growth rate decreased significantly despite the continuous rise in the number of solar power publications, reaching approximately 8500 by 2020. It should be noted that there is a slight decrease in the number of publications in 2021 due to the time lag in the inclusion of the WoS database.

A distinct increase in the annual average citations (*AAC*_*i*_) during 2001–2009 indicated an increased focus on solar power research based on citations. The first peak in citation impact occurred in 2009, with an *AAC*_*i*_ index of approximately 6.76. The index value then declined until a second peak in 2014 of approximately 5.68. Subsequently, the annual number of citations per publication has remained stable. The publications from 2020 and 2021 may have a shorter citation window (fewer than three years) and thus fewer citations. Despite fluctuations, solar power research has witnessed increased academic attention over the last decade.

### Prolific countries and institutions

**Fig. 3:**
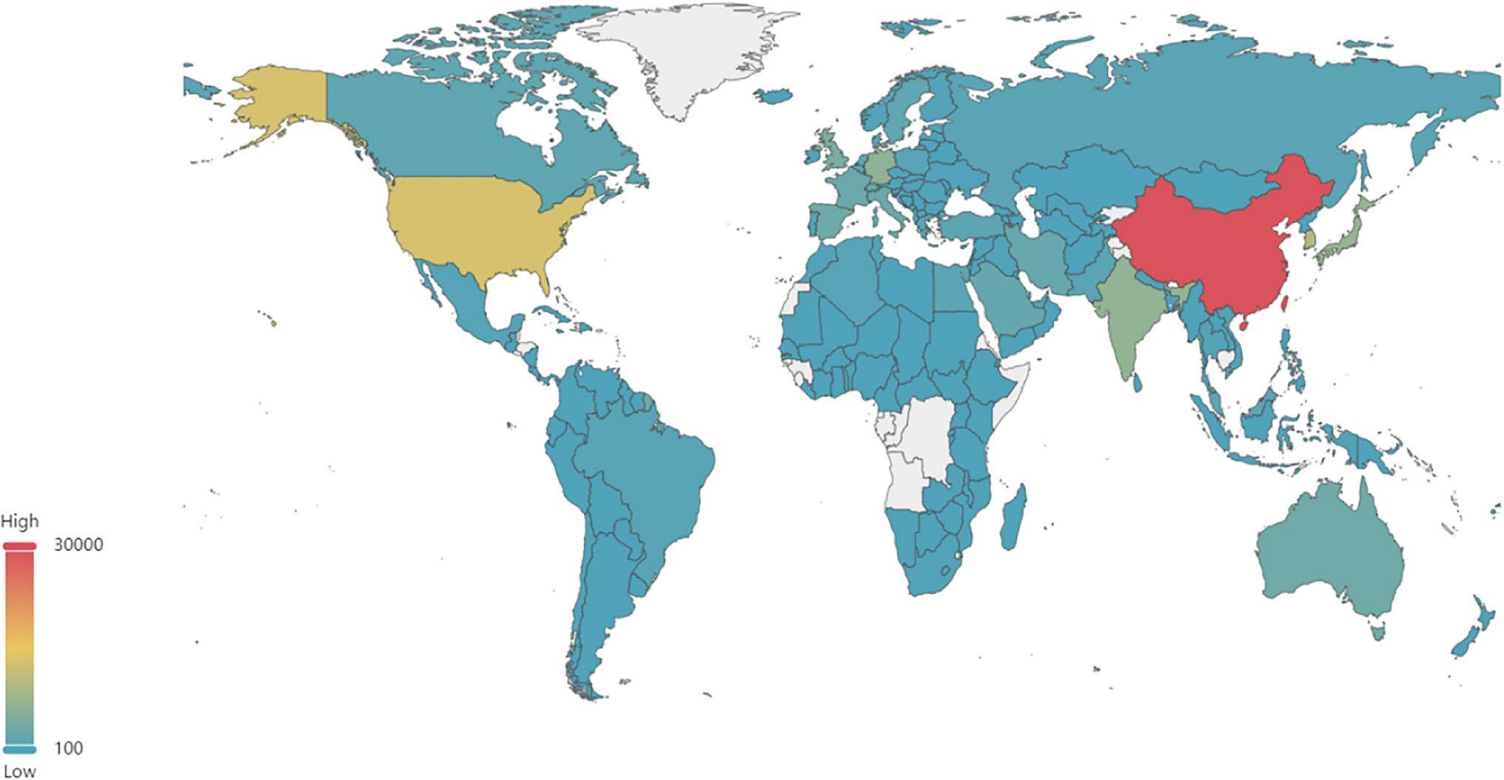
Total number of publications in the dataset for each country globally. The color of each country on the map correlates with its number of publications; red indicates the largest total number of publications, and blue indicates the smallest total number of publications

Full counting was used to calculate the scientific output of a given country, region, or institution. This assigned equal weight to all publications containing an author address from the specific country or institution under consideration, regardless of the frequency of occurrence. Full counting provided non-additive statistics, such as the total number of publications from individual countries or regions exceeding the total global publication count (Waltman 2016). Figure 3 depicts the total scientific output of different countries on solar power generation worldwide in distinct colors. China and the USA emerged as the leading countries for publications in this field.

Table 2 displays the top 10 most prolific countries and regions in solar power research for the period 2001–2021 in detail. Together, these countries and regions have contributed 76.5% of the global solar power papers. Certain Asian and European countries are global leaders in related research. Of the nine countries in the top 10 list, excluding the USA, four are in Asia, and five are European. China ranked the highest with approximately 30,000 publications, more than double the second-ranked USA. Chinese publications accounted for a substantial contribution of over 30% of global publications. The USA accounted for 13.6% of all publications worldwide. South Korea, Japan, and India ranked third to fifth, respectively, with a total output exceeding 6500 publications. These five countries were considered the core contributors to the development of solar power research. Other countries that contributed significantly included Germany, Taiwan (China), the UK, Australia, and Spain, with global shares ranging from 3.3 to 6.6%. These countries were prominent in solar power generation research and featured as the leading producers of solar power worldwide. According to the *Solar Industry Update Report* (Feldman and Margolis 2019), China, India, the USA, Japan, and Europe were among the most significant countries with the highest solar power generation capacity globally.

**Table 2:** Output of the top 10 most prolific countries or regions

Country	*TP*	*TP%*	*ICP%*	*CP%*	*Top-10%*	*AC*
China	29,315	30.7	27.1	97.2	19.9	38.1
USA	13,011	13.6	50.1	98.4	30.2	79.4
South Korea	9843	10.3	27.5	96.0	14.9	37.3
Japan	7134	7.5	33.4	96.5	15.9	45.0
India	6595	6.9	33.7	96.6	9.9	21.8
Germany	6320	6.6	53.5	97.9	22.2	51.8
Taiwan (China)	4589	4.8	22.6	96.8	12.9	35.1
UK	4427	4.6	69.3	98.4	28.0	69.0
Australia	3236	3.4	64.2	98.0	26.0	51.7
Spain	3175	3.3	56.9	97.7	20.3	46.7

International collaboration has become a vital mode of academic research. Compared to Asian countries, developed regions like the European Union (EU) and the USA were more inclined toward participating in international collaborations. Table 2 shows the proportion of international co-authored publications (*ICP%*) for the UK, Australia, Spain, Germany, and the USA which exceeded 50%, while the proportion in China, South Korea, and Taiwan was less than 30%, and the proportion in Japan and India was slightly above 30%. Research from the top 10 countries has received widespread attention. More than 96% of all publications have received citations in each country, with the share of the top 10% highly cited publications ranking above the global average. Among these countries, the USA performed the best in citation impact, with the percentages of cited and top 10% highly cited publications far higher than those of the other countries, and its average citation per publication (*AC*) ranked first (79.4). The UK was also a representative of outstanding academic influence. Conversely, India needed to strengthen its performance for the proportion of highly cited publications (*Top-10%*) and overall citation impact (*AC*).

**Fig. 4:**
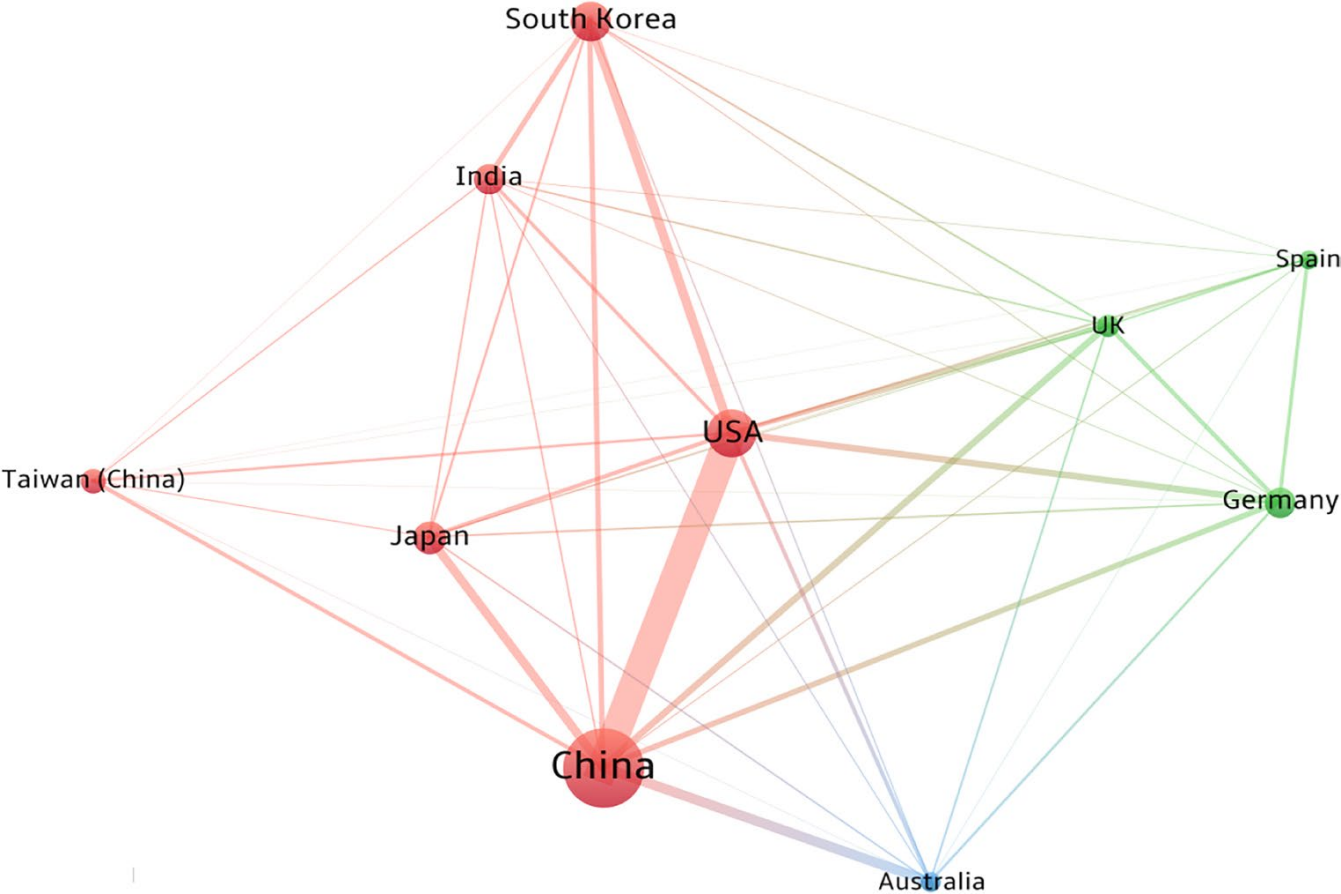
Collaboration network for the top 10 most prolific countries or regions. Each node represents a country or region on the map, and node size depicts the number of publications. The node color represents a cluster, edge connections display collaborations between countries or regions, and thicker lines represent closer collaboration

Figure 4 displays the collaboration network among the top 10 most prolific countries. The dot size indicates the total number of publications of a country. The intensity of collaboration is reflected by the line thickness, where the number of co-authored publications between one country and others determines the total strength of its relations. Figure 4 shows two primary networks that emerged in solar power research. The first was led by China (red cluster) and the second by Germany (green cluster). The USA is located centrally within the network, indicating that it collaborates more with members of both sub-networks and has a clear bridging role. In contrast, Australian collaboration with these leading countries was independent, forming a distinct blue cluster.

Table 3 presents a detailed investigation of the top 10 most prolific institutions globally. Mostly institutions from the USA (3), China (2), and European countries (4), such as France, Germany, and Switzerland, dominate solar power research. Among the institutions engaged in solar power research, the Chinese Academy of Sciences (CAS) significantly outperformed the others in productivity, contributing more than 7% of global publications, which is almost three times that of the second-ranked U.S. Department of Energy (DOE) (2.5%). Following them are the University of Chinese Academy of Sciences from China, Centre National de la Recherche Scientifique (CNRS) from France, and Swiss Federal Institutes of Technology Domain from Switzerland. These three combined accounted for approximately 2.0% of all publications worldwide. Helmholtz Association (Germany), Institute of Chemistry (CAS, China), Ecole Polytechnique Federale de Lausanne (Switzerland), Indian Institute of Technology System (IIT System) (India), and University of California System (USA) ranked fifth to tenth, respectively, with a global share of more than 1.4%. These institutions included national academy, government, nonprofit, academic, and research council or system types implying that solar-related research is an academic issue as well as a policy and social concern. Solar power, as one of the promising renewable technologies, has attracted more attention from the public and government due to the increasing awareness of sustainable development goals.

**Table 3:** Top 10 most prolific institutions worldwide

Organization	Country	Type	*TP (TP%)*	*ICP%*	*CP%*	*Top-10%*	*AC*
Chinese Academy of Sciences (CAS)	China	National Academy	**6983 (7.3)**	23.0	98.4	25.1	49.3
United States Department of Energy (DOE)	USA	Government	2424 (2.5)	44.7	99.1	35.9	99.2
University of Chinese Academy of Sciences, CAS	China	Academic	1890 (2.0)	17.4	98.5	27.3	56.2
Centre National de la Recherche Scientifique (CNRS)	France	Research Council	1826 (1.9)	61.4	97.7	13.6	35.0
Swiss Federal Institutes of Technology Domain	Switzerland	Government	1797 (1.9)	**75.0**	**99.4**	**43.1**	137.1
Helmholtz Association	Germany	Nonprofit	1693 (1.8)	50.4	98.1	21.1	42.4
Institute of Chemistry, CAS	China	Research Institute	1638 (1.7)	24.1	**99.5**	37.0	83.6
Ecole Polytechnique Federale de Lausanne	Switzerland	Academic	1467 (1.5)	**77.2**	**99.4**	**46.8**	**155.4**
Indian Institute of Technology System (IIT System)	India	Academic System	1398 (1.5)	27.1	97.5	10.5	24.4
University of California System	USA	Academic System	1374 (1.4)	55.6	99.3	**43.2**	**159.2**

For international collaboration and citation impact, institutions in the USA and European countries performed better than their Asian counterparts. Almost all the USA and European institutions had an *IPC%* index of over 50%, whereas those of the Asian institutions remained below 30%. Among the top 10 institutions, the Ecole Polytechnique Federale de Lausanne and the Swiss Federal Institutes of Technology Domain, both in Switzerland, showed the highest degree of internationalization, with approximately 80% of their publications being co-authored by researchers from different nations. Similarly, the CNRS of France was distinctly inclined toward international collaboration, with an *IPC%* value exceeding 61%. In addition, the Ecole Polytechnique Federale de Lausanne, Swiss Federal Institutes of Technology Domain, and University of California System demonstrated superior academic influence compared to other institutions. The Ecole Polytechnique Federale de Lausanne had the highest percentage of publications in the top 10% tier (*Top-10%*), while the University of California System received the highest number of citations per publication (*AC*). Despite having the highest proportion of publications being cited (99.5%), the CAS performed moderately well in other citation-based indicators.

### Main subject categories and journals

**Fig. 5:**
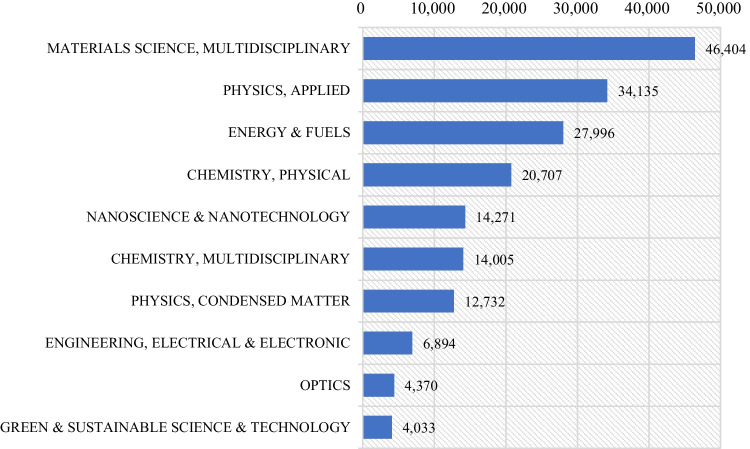
Number of publications (*TP*) of the top 10 most used subject categories

A total of 212 WoS subject categories were relevant to research on solar power generation. The top 10 subject categories with the greatest number of publications from 2001 to 2021 are displayed in Fig. 5. The subject category ranked first, Multidisciplinary Materials Science, had a total of 46,404 publications, accounting for 48.5% of all publications related to solar power generation. Following this category was Physics-Applied (34,135), Energy and Fuels (27,996), and Chemistry-Physical (20,707), which in combination comprised approximately 30%. The third tier included Nanoscience and Nanotechnology (14,271, 14.9%), Chemistry-Multidisciplinary (14,005, 14.6%), and Physics-Condensed Matter (12,732, 13.3%), each comprising more than 10% of publications. Engineering-Electrical and Electronic (6894, 7.2%), Optics (4370, 4.6%), and Green and Sustainable Science and Technology (4033, 4.2%) were the remaining subject categories.

**Fig. 6:**
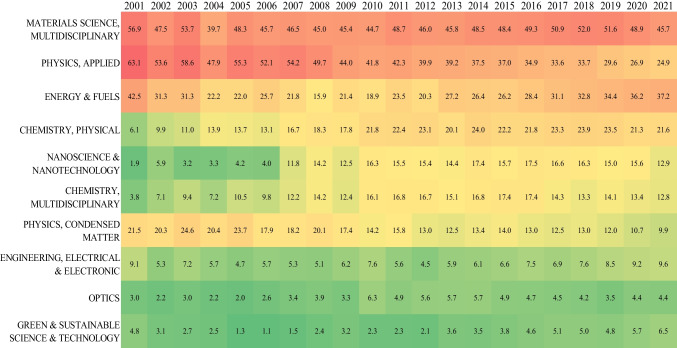
Annual proportion of publications (*TP%*) of the top 10 most used subject categories. The color of each cell in the graph correlates with the percentage of publications; red indicates the highest percentage of publications and green the lowest

According to the dynamic evolution shown in Fig. 6, the Applied Physics subject category demonstrated a publication output advantage during the early stages of the investigation. However, this category was superseded by Multidisciplinary Materials Science in 2008. Subsequently, Multidisciplinary Materials Science has been the foremost category, reflecting a stable increment annually. By 2017, publications within Multidisciplinary Materials Science had exceeded 50% with 4060 published documents. In contrast, there has been a noticeable decrease in the proportion of publications in Applied Physics. Compared to other categories, Condensed Matter Physics has witnessed a decline in the proportion of publications despite an increase in the total number. The outcomes of this investigation confirm, to a certain degree, that increased attention has been focused on foundational disciplines, such as chemistry and materials, within the large-scale implementation phase of solar power technologies (Dong et al. 2012).

Several core journals contributed a significant percentage of publications. Table 4 below lists the top 10 journals with the greatest number of published publications.

**Table 4:** Top 10 journals with the most publications in JCR

Rank	Journal	Quartile	*TP*	*TP%*
1	*Solar Energy Materials and Solar Cells*	Q1	4495	4.70
2	*Solar Energy*	Q2	3228	3.38
3	*ACS Applied Materials & Interfaces*	Q1	2638	2.76
4	*Journal Of Materials Chemistry A*	Q1	2492	2.61
5	*Applied Physics Letters*	Q2	2328	2.43
6	*Thin Solid Films*	Q3	1737	1.82
7	*Journal Of Physical Chemistry C*	Q2	1731	1.81
8	*Organic Electronics*	Q2	1548	1.62
9	*RSC Advances*	Q2	1495	1.56
10	*IEEE Journal of Photovoltaics*	Q2	1457	1.52

*Solar Energy Materials and Solar Cells* ranked first with 4495 publications, constituting approximately 4.7% of all publications in the dataset. *Solar Energy* ranked second with 3228 publications (3.38%), followed by *ACS Applied Materials & Interfaces*, *Journal of Materials Chemistry A*, and *Applied Physics Letters.* All three journals had a proportion of over 2% publications each. The remaining five journals, ranked from sixth to tenth, contributed more than 1.52%. These included *Thin Solid Films*, *Journal of Physical Chemistry C*, *Organic Electronics*, *RSC Advances*, *and IEEE Journal of Photovoltaics*. Our findings suggested that solar power research has attracted substantial attention from researchers in energy, materials, and physics. Additionally, the relatively large proportion (90% = 9/10 × 100) of journals in Q1 and Q2 illustrated the high quality of publications on solar power research. This contributed to improving its dissemination and impact throughout the academic community to a certain extent.

### Hot topics and issues

#### Hot topics indicated by high-frequency title terms

Titles play a crucial role in academic genres, offering a concise overview of significance (Hyland and Zou 2022). Terms within publication titles are indicators to identify current hot topics using bibliometric analyses (Katsurai and Ono 2019; Lyu and Costas 2022). This study employed high-frequency title terms to explore hot topics and their evolutionary trends in solar power research. VOSviewer is utilized to generate, cluster, and visualize the network of title terms from all publications in our dataset, employing a specific text mining approach (Van Eck and Waltman 2011) and a unified framework for mapping and clustering (Van Eck et al. 2010a, b; Waltman et al. 2010). In addition, the overlapping visualization option in VOSviewer enabled further analysis of the evolution process (reflected by average publication year) and the degree of academic attention (reflected by normalized citations) of these research topics on a base map.

**Fig. 7:**
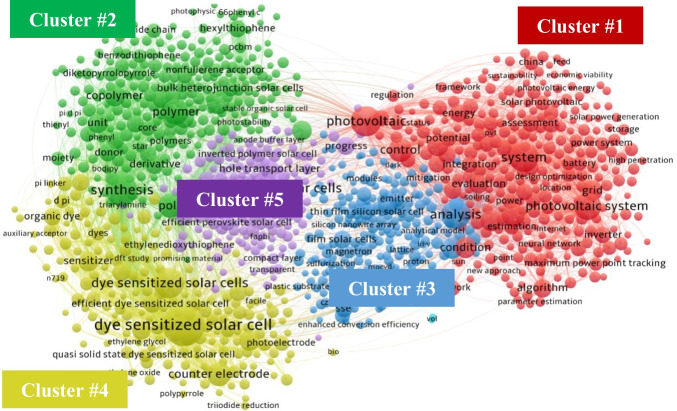
Clusters of high-frequency title terms of publications. Each node represents a title term on the map, and node size depicts the number of publications containing the term. The color of the nodes represents a cluster, and the edge connections show the frequency of co-occurrence between terms in the titles. The thickness of connecting lines represents the frequency of co-occurrence of the terms in titles

Figure 7 presents the clustering network (the base map) of more than 2600 high-frequency title terms (occurrence ≥ 20) based on their co-occurrence relations in publications, providing an overview of current hot research topics on solar power generation. Each item in the figure represents a term extracted from all the publication titles. The size of an item indicates the number of publications containing the corresponding title term, while the thickness of a line is directly proportional to the frequency of co-occurrence between two items. The color of an item represents the main cluster to which it belongs, and the distance between two items offers an approximate indication of the relatedness of their co-occurrences (Van Eck and Waltman 2017). Five clusters with different colors (green, blue, red, yellow, and purple) were identified via the special algorithm in VOSviewer. The list of the top 20 high-frequency title terms for each cluster is shown in Table 5.

**Table 5:** High-frequency title terms in each cluster (top 20)

Cluster	Title terms (in descending order of frequency)
#1	Photovoltaic; System; Photovoltaic System; Control; Model; Photovoltaic Module; Review; Condition; Grid; Evaluation; Algorithm; Module; Energy; Assessment; Case Study; Technology; Generation; Inverter; Photovoltaic Panel; Cost
#2	Synthesis; Polymer; Poly; Polymer Solar Cells; Copolymer; Unit; Circuit Voltage; Bulk Heterojunction Solar Cell; Acceptor; Derivative; Donor; Hexylthiophene; Small Molecule; Photovoltaic Properties; Bulk Heterojunction Solar Cells; Molecule; Electron Acceptor; Thiophene; Side Chain; Core
#3	Analysis; In Ga; Modeling; Film Solar Cells; SSE; Crystalline Silicon Solar Cell; Thin Film Silicon Solar Cell; Cu2znsn; Emitter; Modelling; Reflector; Detection; Light Trapping; Window Layer; Screen; Triple Junction Solar Cell; Silicon Thin Film Solar Cell; Texture; Magnetron
#4	Dye Sensitized Solar Cell; Dye Sensitized Solar Cells; Counter Electrode; Photoanode; Sensitizer; Electrolyte; Complex; Efficient Dye Sensitized Solar Cell; Nanotube; Organic Dye; Group; Solid State Dye Sensitized Solar Cell; Photoelectrode; Dye Sensitized Solar Cell Application; Triphenylamine; DSSC; Quantum Dot Sensitized Solar Cells; D Pi; Dyes; Theoretical Study
#5	Perovskite Solar Cells; Efficient; Electron Transport Layer; Hole Transport Layer; Progress; Hole Transporting Material; Hole Transport Material; Efficient Perovskite Solar Cell; Planar Perovskite Solar Cell; Inverted Polymer Solar Cell; Stable Perovskite Solar Cell; Stable Perovskite Solar Cells; Highly Efficient; Hysteresis; Interface Engineering; Planar Perovskite Solar Cells; MAPBI; Ethylenedioxythiophene; Perovskite Film; Efficient Perovskite Solar Cells

Cluster #1 (in red) included numerous items and linkages, primarily focusing on PV systems from a system level. The research emphasized grid integration, control, and optimization of solar cell systems. Rather than focusing on individual components, the cluster explored topics, such as maximum power point tracking (MPPT) technology and the versatility of PV systems. Furthermore, there was a focus on economic feasibility, with studies aiming to provide design options to maximize benefits based on empirical case studies. Government and public interest in solar power technologies, along with their prioritization of costs and benefits, contributed to the significance of this cluster.

Cluster #2 (in green) is predominantly focused on organic solar cells, particularly acceptors and organic electronics. The cluster emphasized polymer-fullerene bulk heterojunction solar cells, considered among the most promising types of organic solar cells, and attracted considerable attention from researchers. However, despite their high flexibility and mobility, these devices exhibited lower efficiency compared to other types due to their structure. Researchers in this cluster were actively exploring various materials and solutions to enhance the blending between acceptor and donor, aiming to optimize the configuration of the cells and achieve higher efficiency. Additionally, diverse schemes and strategies for designing cells were evaluated to improve overall performance.

Cluster #3 (in blue) focused on film solar cell modules, including various technologies that used different materials. Recently, copper indium gallium di-selenide emerged as a focus within this cluster. Additionally, research in this field investigated innovative approaches, including light trapping, Lambertian reflectors, and surface texturing. The nodes within this cluster exhibited a weak interdependence, suggesting less concentrated research activity and a more autonomous exploration of diverse aspects related to film solar cell modules. Cluster #3 demonstrated a close interaction with Cluster #1, particularly concerning system evaluation.

Cluster #4 (in yellow) focused on dye-sensitized solar cells (DSSCs). DSSCs are a hot topic and categorized as excitonic solar cells, focusing on organic dyes like triphenylamine. However, DSSCs remained in the nascent stages of development, and enhanced efficiency is a primary objective. Researchers within this cluster are exploring different materials for the integration and measurement of each component, including quantum dots for high light stability, solid-state electrolytes for better temperature response, and modified doping of TiO_2_ to match the electrolytes used, such as carbon nanotubes and ZnO. Nonetheless, most investigations remained theoretical, requiring additional time for commercialization. Cluster #4 demonstrated close interaction with Cluster #1, particularly for organic dyes.

Cluster #5 (in purple) studied perovskite PV solar cells, focusing on modifying and comparing layer properties. Although this cluster included the fewest high-frequency title terms, the dense proximity of nodes attested to a high level of relevance. Hole-transporting layers (HTLs) constituted an essential component of perovskite PV solar cells, typically formed with polymer materials, such as P3HT, PTTA, and PEDOT, which Cluster #5 explored. This was also relevant to Cluster #4, particularly concerning electron transport and diffusion concerns. Research into novel organic materials for enhancing the performance of HTLs was a significant focus within this cluster. Cluster #5 had a strong interaction with Cluster #1, particularly through the examination of system performance.

Third-generation PV cells and their related components are currently the dominant focus in this field. The solar cell types listed in Clusters #2–#5 belong to the third-generation category. Additionally, the growing interest in building-integrated photovoltaics (BIPV) has led researchers to explore system performance.

### Evolution of hot topics over time

**Fig. 8:**
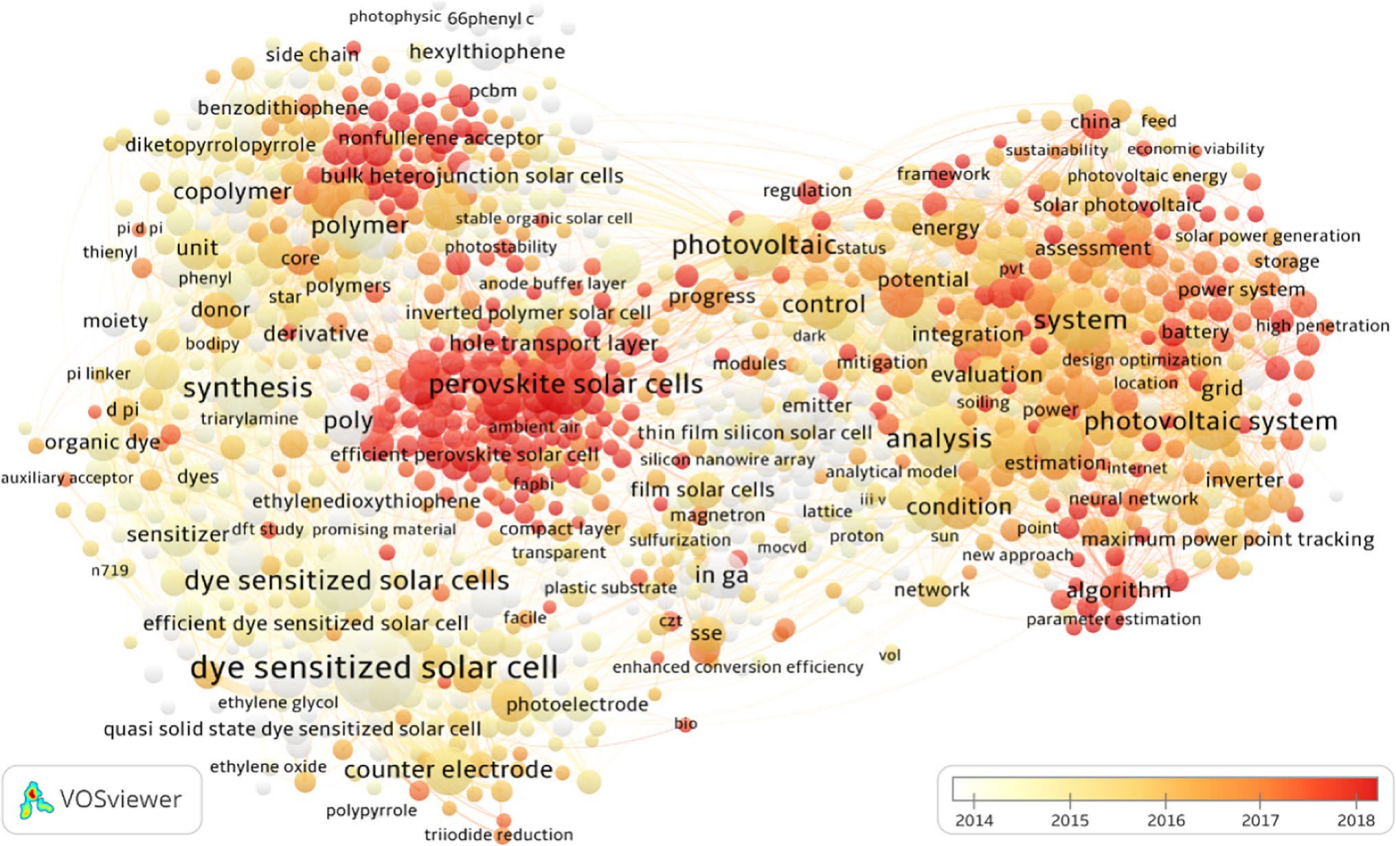
Average publication year of each title term. The color coding of each item is determined by its overlay score, i.e., the average publication year of all the publications containing the term. Darker colors indicate a later average publication year and a higher degree of novelty

Figure 8 complements the insights gained from Fig. 7 into the evolution of research hotspots over time by overlaying the base map of the average publication year of each title term. The color coding of each item was determined by its overlay score, representing the average publication year. Darker shades indicate a later publication year, suggesting a higher degree of novelty. The color progression was from the bottom left to the top right and middle, corresponding to clusters #2, #4, and #3, and #1 and #5, respectively, with clusters #1 and #5 representing relatively newer topics. While clusters #2 and #4 have darker dots, a significant proportion of these belong to the period before 2015 (depicted in white and yellow). Before 2013, the center of related research was likely cluster #2, which housed nearly all frequent title terms from that period. From 2016, the research focus shifted to the other three clusters, and the predominance of cluster #4 emerged. Subsequently, starting in 2018, clusters #1 and #5 began to house most topics, with cluster #5 representing the emerging field.

Research on solar power generation over the last two decades has predominantly focused on third-generation solar cells, as illustrated in Fig. 8. This inquiry commenced with investigations into organic solar cells, dye-sensitized solar cells, and thin-film solar cells, with the bulk of research being published before 2015. During this period, the solar power system was still in its nascent stage, and the solar cell was not sufficiently developed to function independently. However, with the growing advancement of materials, diverse kinds of solar cells emerged, and the solar power system gradually became recognized as an autonomous energy system. As the systems matured after 2015, they were subjected to increasing demands, especially for fulfilling performance and grid integration targets, leading to frequent optimization and efficiency improvements. Subsequently, from 2016 onwards, research on these new technologies began to dominate, with perovskite PV solar cells emerging as the benchmark after 2018. Research on hole transport layers (HTLs) is currently one of the most compelling emerging areas of inquiry.

Another topic is non-fullerene acceptors in organic solar cells, which have the potential for record power conversion efficiencies and improved chemical, thermal, and photostability. Research has evolved to consider socio-economic impacts alongside traditional technical performance analyses in system-level investigations. Considering sustainability goals and the increasing demand for electricity, solar power generation is predicted to expand significantly in future years. However, intermittency and instability remain significant bottlenecks, prompting researchers to explore the feasibility of battery integration and control algorithm optimization. Initial findings have yet to produce conclusive results. Finally, while research on the newest areas of inquiry remains small in scale and is yet to produce significant, published results, it is essential to the exploration stage. Conversely, the large dots represent long-term research topics that have been accumulated more publications over a longer period and are more established.

The combination of Figs. 7 and 8 revealed that the primary challenge in solar PV technology is the low efficiency in converting sunlight into electricity. Despite an efficiency increase attributed to the development of novel materials, the total numbers have remained low, with a maximum efficiency of 26% observed in laboratory conditions. This outcome was particularly notable in perovskite solar cells, which have attracted significant attention since 2018. Over the past 3–5 years, material development has stagnated, resulting in consistent efficiency levels despite the growing number of published articles. Discovering or producing a suitable material in the short term may pose a challenge. Consequently, developing coating layers for perovskite solar cells becomes a new focal point to enhance performances over the next several years.

Additionally, systematic analysis is crucial for PV applications, with power capacity as a key indicator. Rather than relying on rough assessments using annual or monthly data, hourly time steps with advanced algorithms offer a more accurate and representative output. Considering the sensitivity of PV power generation to weather conditions, further research is warranted to predict future power capacity under the influence of climate change. Lastly, as the lifespan of the initially installed PVs nears its end, finding a solution for their disposal, rather than resorting to current landfill practices, has become urgent. The recycling treatment of materials from these PVs will emerge as a prominent research area in the future.

### Citations of hot topics and highly cited publications

**Fig. 9:**
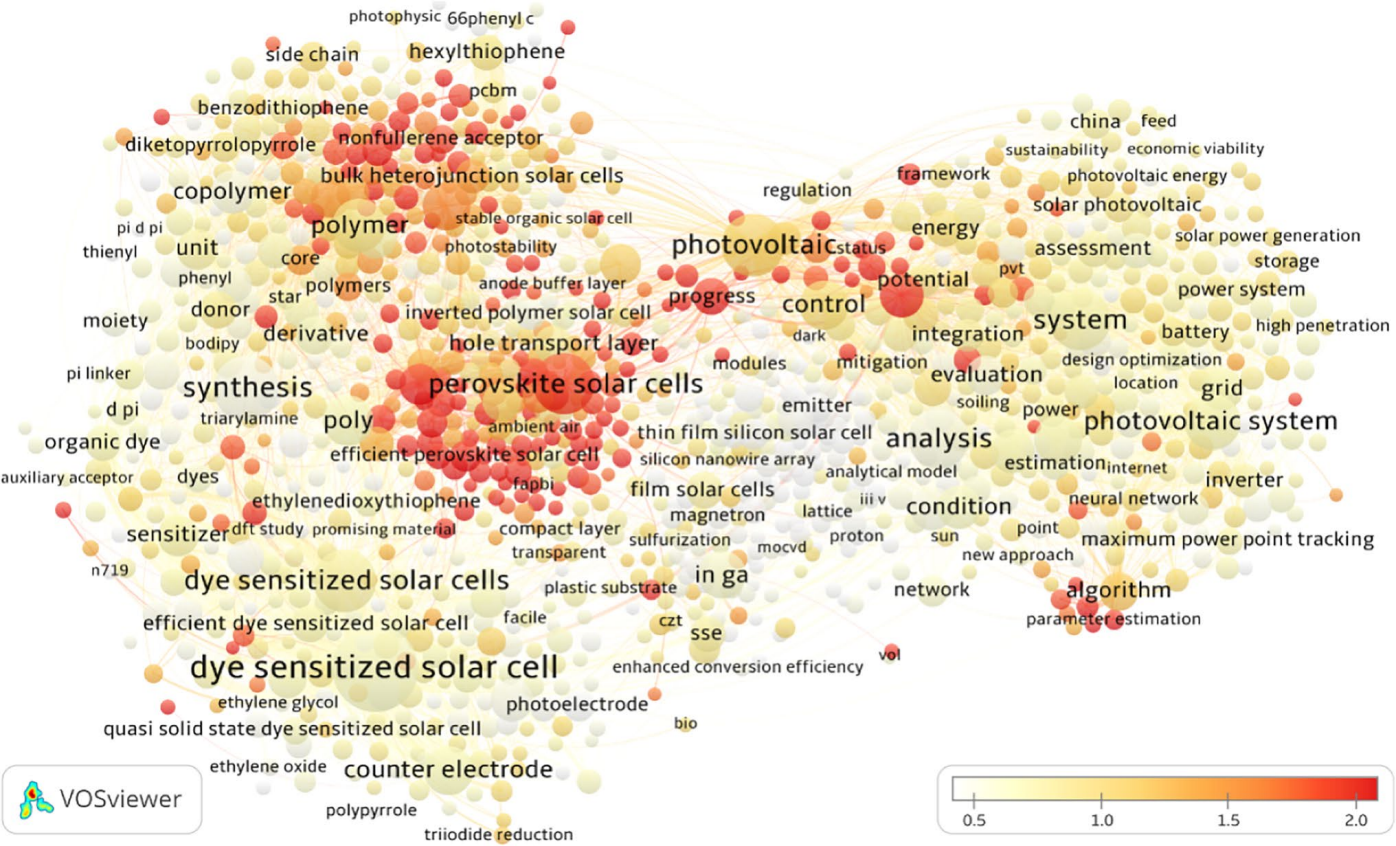
Average normalized citations (The citation score used on the map is normalized by dividing by the mean. Thus, if the score of a title term exceeds 1.00, the average citation impact of publications containing that term exceeds the average.) for each title term. The color coding of each item is determined by its overlay score, i.e., the average category normalized citation index (*CNCI*) of each term. Dark colors indicate higher average normalized citations and higher academic attention

Figure 9 illustrates the average category normalized citation index (*CNCI*)^2^ of each title term in the base map, depicting the main academic focus of topics based on citations. The visualization used a color scale ranging from white and yellow to red, with darker colors signifying higher average normalized citation values and greater academic impact. Similar to Figs. 8 and  9 demonstrated a consistent trend toward darker colors from bottom left to top right, originating in clusters #2, #4, and #3 and culminating in clusters #1 and #5. Cluster #2 consistently corresponded to the lightest color, indicating the fewest citations. Conversely, clusters #1 and #5 exhibited significant advantages in citation impact, represented by the prevalence of darker points. Despite this, the topics attaining the highest number of citations did not necessarily correlate with the volume of publications. Novel topics with smaller sizes tended to attract greater attention from researchers.

Solar power research predominantly focused on individual types or components rather than a comprehensive system. These solar cells faced numerous challenges, such as high costs, low efficiency, and potential pollution during manufacturing processes, necessitating innovative materials, components, and schemes. Current research primarily focuses on perovskite solar cells and non-fullerene acceptors. Considering the potential of solar power generation as a renewable option for future electricity networks, the field continued to attract significant interest from both academia and the public. The visual representation of the research landscape revealed that while larger dots attract attention, moderately smaller ones entail hot topics in established fields. The mature aspects, such as dye-sensitized solar cells, tend to receive less attention due to the abundance of preexisting literature, which, in turn, facilitates interest in uncharted areas.

Table 6 lists the top 10 most cited publications in our dataset. The analysis of these highly cited publications reveals common characteristics. Firstly, most of these publications were published around 2010 and have a lengthy citation window. Secondly, these publications were published in prestigious journals known for their wide dissemination and wide readership. Thirdly, the research topics of these publications largely center around technologies or materials for improving the performance of solar cells. This focus has resulted in high visibility and significant follower bases.

**Table 6:** Top 10 publications with the most citations in the WoS database

	Title	PY	Type	Journal	*TC*
1	Organometal halide perovskites as visible-light sensitizers for photovoltaic cells	2009	Article	*Journal of the American Chemical Society*	14,071
2	Efficient hybrid solar cells based on meso-superstructured organometal halide perovskites	2012	Article	*Science*	7982
3	Sequential deposition as a route to high-performance perovskite-sensitized solar cells	2013	Article	*Nature*	7450
4	Dye-sensitized solar cells	2010	Review	*Chemical Reviews*	7266
5	Plasmonics for improved photovoltaic devices	2010	Review	*Nature Materials*	6573
6	Lead iodide perovskite sensitized all-solid-state submicron thin film mesoscopic solar cell with efficiency exceeding 9%	2012	Article	*Scientific Reports*	6198
7	Efficient planar heterojunction perovskite solar cells by vapour deposition	2013	Article	*Nature*	6187
8	Conjugated polymer-based organic solar cells	2007	Review	*Chemical Reviews*	5547
9	Interface engineering of highly efficient perovskite solar cells	2014	Article	*Science*	5282
10	Porphyrin-sensitized solar cells with cobalt (ii/iii)-based redox electrolyte exceed 12 percent efficiency	2011	Article	*Science*	5219

## Conclusion

Solar energy, a principal renewable energy source, has attracted significant attention from the global academic community over the past two decades. This publication provides an up-to-date overview of the development of solar power research over the past 20 years on a global scale, using bibliometric methods and visualization techniques. The main conclusions are as follows:

1. From 2001 to 2021, the annual output of global publications on solar power generation has increased significantly, especially since 2009. Given the growing prominence of energy-related concerns, interest among academic researchers has increased regarding this topic. Despite fluctuations in the *AACi* index and academic impact, solar power research has gained widespread attention in the last decade. The rapid growth of solar power research is largely due to its socio-economic and political significance. Solar power research will continue to thrive, given the pressing demand for new energy sources. These trends underscore the constant evolution in solar power generation globally and the ongoing efforts to promote sustainable practices and address pertinent issues related to energy access and affordability.
2. Asian and European countries are the global leaders in solar power research. China emerged as the leading contributor, accounting for over 30% of all publications, followed by the USA. South Korea, Japan, and India rank third to fifth. These five countries are the core contributors to the development of solar power research. Additionally, Germany, Taiwan, the UK, Australia, and Spain demonstrate noteworthy scientific output. Compared to Asian countries, developed countries like the EU and the USA were more inclined to international collaborations. Certain core institutions, CAS significantly outperformed the others in productivity, while the Ecole Polytechnique Federale de Lausanne and Swiss Federal Institutes of Technology Domain rank higher in academic impact.
3. Solar power generation has attracted considerable attention from researchers across several subject categories, including fundamental areas of study. The five primary subject categories in related research were Multidisciplinary Materials Science, Applied Physics, Energy and Fuels, Physical Chemistry, and Nanoscience and Nanotechnology. Journal analysis confirms that energy and physics researchers are increasingly interested in solar power research. Prominent journals include *Solar Energy Materials and Solar Cells*, *Solar Energy*, *ACS Applied Materials & Interfaces*, *Journal of Materials Chemistry A*, and *Applied Physics Letters*, which have published substantial numbers of solar power-related research, providing an essential platform for academic discourse. The relatively significant proportion of journals classified in Q1 and Q2 illustrates the high quality of publications on this topic which can be attributed to the enhanced dissemination and impact of achievements in solar power generation research.
4. In the last two decades, researchers have directed their attention toward improving the efficiency and enhancing the stability and flexibility of third-generation solar cells. Current research efforts focus on novel materials and devices to meet increasing technical and economic requirements. Additionally, optimizing system operations is a pertinent area due to the development of related technologies. From an academic standpoint, the current areas of inquiry are similar to those within the emerging field, but they are more targeted and concentrated on specific aspects, such as non-fullerene, transport hole layers, and algorithms. Highly used terms, such as “acceptor,” “perovskite solar cell,” and “algorithm,” are valuable topics of future research, as evidenced by their consistently higher average publication year and normalized citation. In addition, another two areas of interest are emerging: capacity prediction under the influence of climate change and the recycling management of retired PVs.

## Further discussion

Despite certain limitations, including excluding the humanities and social sciences research, the findings provide valuable data for researchers, policymakers, the public, and newcomers.

The main contributors (i.e., countries and institutions), science landscape (i.e., subject categories and journals), and hotspots (i.e., high-frequency title terms) of global solar power research are identified. Solar power has emerged as one of the most promising renewable technologies, demonstrating substantial progress over the past two decades. These advancements include efficiency improvement, system integration, and cost reduction. Awareness of these advancements provide guidance newcomers and enhance awareness of technological developments in this area.

Future focal points for researchers and institutions in the solar power domain will include new materials with higher efficiency and greater commercial availability and implementing improved operation strategies that integrate grid and storage systems. Solar power research is vital in supporting this emerging field, achieving global sustainable development goals, such as carbon neutralization, and promoting collaboration among researchers from different domains. The growing demand has resulted in an influx of new talent and investors. Hence, long-term growth will be sustained. This study demonstrates that solar power has made significant progress and highlights areas that require further attention to continue its growth trajectory.

## Data Availability

Data will be made available upon request.

## Footnotes

1 https://apps.webofknowledge.com/

2 The Category Normalized Citation Index (*CNCI*), developed by Clarivate, is a normalized citation indicator calculated by dividing the citation counts received by a publication by the average citations garnered by publications published within the same research field, document type, and publishing year.

## Declarations

### Data transparency

The lead author confirms that the manuscript is an authentic, precise, and transparent report of the study being reported.

The authors confirm that this manuscript has not been published elsewhere and is not under consideration by another journal or has not been submitted to a preprint server before submission on ESPR.
